# Knowledge, attitudes, and practices of public secondary school teachers on Zika Virus Disease: A basis for the development of evidence-based Zika educational materials for schools in the Philippines

**DOI:** 10.1371/journal.pone.0214515

**Published:** 2019-03-28

**Authors:** Ernesto R. Gregorio, John Robert C. Medina, Marian Fe Theresa C. Lomboy, Andre Dominic P. Talaga, Paul Michael R. Hernandez, Mitsuya Kodama, Jun Kobayashi

**Affiliations:** 1 Department of Health Promotion and Education, College of Public Health, University of the Philippines Manila/SEAMEO-TROPMED Regional Center for Public Health, Hospital Administration, and Environmental and Occupational Health, Manila, Philippines; 2 Department of Environmental and Occupational Health, College of Public Health, University of the Philippines Manila/SEAMEO-TROPMED Regional Center for Public Health, Hospital Administration, and Environmental and Occupational Health, Manila, Philippines; 3 Department of Global Health, Graduate School of Health Sciences, University of the Ryukyus, Nishihara City, Japan; 4 Japanese Consortium for Global School Health Research, University of the Ryukyus, Nishihara City, Japan; Faculty of Science, Ain Shams University (ASU), EGYPT

## Abstract

The Philippines is at risk in developing a Zika Virus (ZIKV) Disease Outbreak. One of the possible interventions is health education because students are potential health advocates and influencers to their communities through the knowledge transfers from their teachers. The competency of Filipino teachers on ZIKV Disease is yet to be described. This study aimed to assess the knowledge, attitudes, and practices of public secondary school teachers on ZIKV Disease. A modified version of the Knowledge, Attitudes, and Practices Survey Tool on Zika Virus Disease from the World Health Organization—Pan American Health Organization (WHO-PAHO) was used to assess the knowledge, attitudes, and practices of teachers of eight public secondary schools in five villages in Quezon City, Philippines. Out of the 609 respondents, 87.3% reported that their main source of information about ZIKV is tri-media, which includes television, print, and radio. Majority of the respondents mentioned that ZIKV is transmitted through a mosquito bite (80.3%). However, only half of the participants identified vector control as a preventive strategy. Moreover, only 54% admitted to have cleaned their water containers or water sources within the last week. Only a few identified mother-to-child (23%) and sexual intercourse (8%) as other means of transmission. Half (49.8%) of the respondents felt that it is possible to acquire ZIKV in their community, while 90% perceived that a private doctor (90%) or a public hospital (88%) can effectively treat the disease. Perceived stigma is high at 50%. This study showed there was good knowledge among teachers on vector transmission of ZIKV but poor knowledge on other aspects of the disease. Also, there was a low level of perceived susceptibility and severity of ZIKV which can be explained by the absence of a personal encounter with a Zika patient and the low number of cases in the Philippines. Half of the respondents said that they cleaned the possible mosquito breeding sites within the last week, followed by those who cleaned their water source more than a week ago (19%). None of the socio-demographic characteristics is significantly associated with respondents’ knowledge on Zika. Only income and location of residence were found to be significantly associated with attitudes towards Zika. These findings call for a comprehensive training program that includes development of teaching materials for public secondary school teachers on ZIKV Disease based from this study.

## Introduction

Nowhere has the impact of globalization been more felt than in the last two decades. It has led to a greater mobility of people and goods which, in turn, contributed to an improved global health and development. However, this has also paved the way for efficient disease transmission, resulting in frequent occurrence of outbreaks in the affected country. Among the emerging diseases, Zika Virus (ZIKV) Disease, a vector-borne health problem, has recently become a major international concern in low and middle income countries. ZIKV Disease has similar clinical manifestations with dengue and chikungunya, which include mild fever, headaches, conjunctivitis, maculopapular rashes, arthralgia, myalgia, lymphadenopathy, and retro-orbital pain. It can also cause congenital abnormalities, collectively known as congenital Zika Virus Disease (CZVD). The public concern over ZIKV Disease was compounded by its ability to be transmitted through both sexual and mother-to-child route. The latter can cause microcephaly among affected babies [1].

Zika Virus was first identified in a Ugandan forest following a yellow fever surveillance among rhesus monkeys in 1947. The virus was later isolated from humans in Uganda and the United Republic of Tanzania. This was followed by reports of human infections from across Africa and Asia from 1960s to 1980s. Outbreaks had also been reported in Yap Island, Micronesia [2] in 2007, French Polynesia in 2013, and in the South and Central Americas in 2015 [3]. In September 2016, imported cases had been documented in most Asian countries. Countries in South-East Asia and the Western Pacific Regions such as Malaysia, the Philippines, Singapore, Thailand and Vietnam continued to report new cases of Zika from 2015 to the present [4]. Recently, Zika Virus was detected in Myanmar through analysis of serum samples from 2004 to 2017 [5].

In 2016, the Philippines has been classified by World Health Organization (WHO) under Category 2, which includes countries with possible endemic transmission or evidence of local mosquito-borne Zika infection [4]. A serologic study conducted in the country in 1953 detected 19 Zika-positive samples out of 153 sera that were examined. Decades later, a confirmed case from a prospective cohort of 267 acute febrile patients in Cebu, a province in central Philippines, was reported in March 2012. In September 2016, a confirmed case of Zika Virus infection was reported in Iloilo City, a city in central Philippines, and was followed by two other patients a week later. Three weeks thereafter, cases were confirmed from the cities of Cebu and Muntinlupa (a city in Metro Manila). The case from Cebu City was a 22-year old primigravid of 19 weeks. The case from Muntinlupa City was initially reported in Laguna, a province South of Metro Manila, but the detail was clarified. Two more cases were reported in Iloilo City, and one in the municipality of Oton, Iloilo prior to the end of September. As of February 2017, the Philippine Department of Health had reported 57 Zika cases including seven cases of pregnant women. [6]

The presence of the *Aedes* mosquito in the Philippines and the number of returning Overseas Filipino Workers (OFW) from Zika-endemic countries can pave the way for future outbreaks ([7]. Since Zika virus can be transmitted through sexual intercourse and from mother to fetus, another social concern that may contribute to a higher possibility of establishing an endemic focus in the Philippines is the increasing trend of adolescent pregnancy which may increase the risk of complications in the unborn child [8].

Health education on self-protection from mosquito bites, on cleanliness of the surroundings, and on elimination of breeding sites remains to be the best way to prevent human transmission. School-based health education can complement community health education, since the former provides an appropriate vehicle for a more structured and integrated prevention education program. Several studies have shown that developing students as health messengers or change agents were effective in combatting malaria in Ghana [9] and dengue in Sri Lanka [10].

There had been knowledge, attitudes and practices surveys done on Zika Virus among medical doctors [11], pregnant women [12, 13], university students [14, 15, 16] and community residents [17,18, 19] in different countries but none among secondary school teachers. Teachers can serve as reliable sources of health information that can improve the literacy of school-based adolescents on Zika infection [20]

Improving the knowledge, attitudes, and practices of teachers on diseases such as Zika is important because they can transfer these competencies to their students who can eventually diffuse the knowledge gained to their respective families and communities [9, 10]. The present knowledge, attitudes, and practices of Filipino teachers on ZIKV Disease is yet to be described. Such baseline data can serve as a basis for the development of evidence-based health education materials and modules about Zika Virus that can be used by teachers in the schools. It can also be used to assess the effectiveness of future school-based health education interventions towards ZIKV Disease prevention and control in the future. Hence, this study aimed to determine: 1) the knowledge of Philippine public secondary school teachers on ZIKV Disease, its modes of transmission, signs and symptoms, prevention and control; 2) their attitudes towards ZIKV Disease and its importance as a psychosocial issue; and 3) their existing health practices related to the prevention and control of ZIKV Disease.

## Methods

### Research design

The study used a descriptive cross-sectional study design to assess the knowledge, attitudes and practices of public secondary school teachers on Zika Virus Disease.

### Study site

The study was done in selected public secondary schools in Quezon City (QC), which is composed of 143 villages. The study site was purposively selected because seven of its villages were listed among the dengue hotspot areas National Capital Region in 2015, i.e. with yellow alert level. Clustering of at least three cases in the past four consecutive weeks placed a village into yellow alert level [21, 22]. Among the seven villages, villages 1, 2, 4, 5, and 6 were considered during the first selection of study sites due to their accessibility and lower security concerns. The schools situated in these villages were identified using the official list of public secondary schools in QC as the sampling frame. Invitation to participate in the survey was sent to the school heads. However, the school heads from villages 4 and 6 declined to participate for unknown reasons which necessitated replacement with another hotspot village. Affirmative responses were obtained from the head of two new schools from village 3. In addition, a school from the village 8, which was considered as a potential study site by virtue of its vicinity with the other hotspot villages, was included upon the approval of the school head. In total, eight schools from five villages were included in the final selection of study sites; i.e. one school each from villages 1, 5, and 8; two from village 3; and three from village 2.

Two out of the ten schools which were invited refused to participate but these were replaced with two new schools from the same study area.

### Study population

The target participants of the study were all the teaching staffs who hold the position of a Teacher, Master Teacher, or Head teacher in the participating schools. Through coordination with the office of the school principal, those who are regular employees were identified and invited to participate. There were a total of 1,184 teachers from the eight participating schools and 625 participated which gave a response rate of 53%. Of the 625 who agreed to participate, 609 provided a complete set of data for analysis.

### Research instrument

The study adapted and used with permission the Knowledge, Attitudes and Practices Survey Tool on Zika Virus Disease that was developed by the World Health Organization—Pan American Health Organization (WHO/PAHO) [23]. A group of experts reviewed the tool in terms of content validity, clarity, and cultural sensitivity. They include a health promotion and education expert (ERG), a medical doctor with global health expertise (JK), an expert on global health and disaster medicine (MK), and another medical doctor with a background on linguistics (JCM).

A Self-Administered Questionnaire (SAQ) was developed with four domains. The first domain included 17 socio-demographic information of the respondents. The second domain is composed of items gauging the respondents’ knowledge on ZIKV Disease, its cause, modes of transmission, signs and symptoms, treatment, prevention, and control. The domain is composed of one open-ended question and 30 pre-coded questions, of which 12 can have multiple answers. The third domain has 19 items that measures the attitudes of the respondents towards ZIKV Disease in terms of treatment, testing, prevention and control and stigma. A four-point Likert scale was used to determine the degree of agreement or disagreement of the participants with the statements. Consisting of one open-ended and 19 pre-coded questions, the last domain focused on the respondents’ practices related to the prevention of ZIKV Disease.

The tool was originally written in English and translated to Filipino by two, independent, native, bilingual Filipino speakers. Then, backward translation was done independently by two native, bilingual Filipino speakers. The research team reviewed the back-translated SAQ and revised as deemed necessary. The final version was pre-tested among 12 teachers in a secondary public school in Manila. A focus group discussion was conducted among the same set of teachers for feedback regarding the clarity and cultural sensitivity of the tool.

Reliability testing was done by including all of the items collectively in the analysis. The overall Cronbach’s α score was 0.755. For knowledge and attitude items, the computed alpha were 0.845 and 0.876, respectively, which are indicative of high internal consistency. The Cronbach’s α remains stable within the range of 0.740–0.750 during the sequential deletion of an item.

### Data collection, management, and analysis

Proper coordination was done during data collection. An endorsement for the study was first obtained from the Schools Division Superintendent of the Department of Education Quezon City Division Office. Permission to conduct the survey in their respective schools was sought from the school principals. In each school, the SAQs were delivered and distributed among the teachers. The respondents were given one to seven days to accomplish the SAQs prior to collection.

Gathered data were encoded using Microsoft Excel 2013 and were analysed using IBM SPSS version 24. Only descriptive statistics was employed in describing the socio-demographic profile of the respondents, as well as their knowledge, attitude, and practices related to ZIKV disease. Mean and proportions were estimated for continuous and categorical variables, respectively. For the attitude domain, the frequency of respondents who exclusively reported strongly agree and agree were collapsed into “Agree”. Similarly, those who responded strongly disagree and disagree were aggregated into “Disagree”. A score of 4 was given to a response that strongly agrees with a positively-phrased statement and a score of 1 to a response that strongly disagrees with such statement. The score was reversed for negatively-phrased statements.

To determine the association between the respondents’ knowledge on ZIKV transmission and the socio-demographic characteristics, two responses to the question “How does a person get Zika?” were purposively selected. These are the mosquito bites and sexual transmission. Teachers can better relate with these modes of transmission and these represent the more likely infection routes during an epidemic as compared to mother to child and through blood transfusion. Participants who were able to correctly identify ‘mosquito bite’ and ‘sexual intercourse’ received one unit point for each response. Hence, the maximum score is two unit points. Percentage score was calculated by multiplying the quotient of the sum of unit score in each item and the maximum score with 100. Upon obtaining the percentage score, each participant was dichotomized into having adequate and inadequate knowledge. The following statements: *‘A pharmacy or local drug vendor can effectively treat a person with Zika’* and *‘If somebody in my family were to get Zika*, *I would want it to remain private or a secret’* were purposively selected to represent negative attitudinal traits of Filipinos which contribute to poor health-seeking behaviors e.g., self-medication and low medical and health facility consults [24, 25]. These negative attitudes, if not addressed, could contribute to disease transmission and potential outbreaks. Those who disagree received 2 unit point for each statement, hence the maximum score is 4. Percentage score was calculated by multiplying the quotient of the sum of unit score in each item and the maximum score with 100. Upon obtaining the percentage score, each participant was classified into having favorable and unfavorable attitude. Favorable attitude towards Zika is operationally defined as having a percentage score of greater than 50%. Socio-demographic data such as age, sex, education, religion, position in the school, monthly household income, location of the household, distance to the nearest health center were dichotomized and served as the exposure variables. To determine if association exists between the socio-demographic variables and knowledge and attitude towards Zika disease, a Pearson Chi-Square Test was done. Fisher’s Exact Test was likewise used for a variable that has at least one cell with less than five expected counts. The level of statistical significance (α) was set at 0.05 with 95% level of confidence.

### Ethical considerations

This study has received both technical and ethics approval from the University of the Philippines Manila, College of Public Health Technical Review Committee (UPM-CPH-TRC) and the University of the Philippines Manila Research Ethics Board (UPMREB), respectively.

## Results

### Socio-demographic profile

There were a total of 609 teachers who agreed to participate in the study. The highest proportion of respondents (25.8%) came from School E. Table 1 shows the number and proportion of respondents per school are distributed is as follows:

**Table 1:** Distribution of respondents according to school where they teach (n = 609).

School	Frequency	%
SCHOOL E	157	25.8
SCHOOL G	87	14.3
SCHOOL F	79	13.0
SCHOOL C	74	12.2
SCHOOL D	63	10.3
SCHOOL A	57	9.4
SCHOOL H	57	9.4
SCHOOL B	35	5.7
**Total**	**609**	**100.0**

Table 2 shows that the age of the respondents range from 20 to 64 years old with a mean of 38.6 years old. The highest proportion (39.6%) belongs to the 30 to 39 age group. There were more female respondents (68.5%) than males.

**Table 2:** Distribution of respondents according to age group and sex (n = 609).

Age group	Female	Male	Total	Percentage
20–29	81 (19%)	23 (17.8%)	105	17.2
30–39	174 (41.7%)	57 (44.2%)	241	39.6
40–49	100 (24%)	31 (24%)	138	22.7
50–59	53 (12.7%)	14 (10.9%)	72	11.8
60 and above	9 (2.2%)	4 (3.1%)	14	2.3
No Response			39	6.4
**Total**	**417 (68.5%)**	**129 (21.2%)**	**609**	**100**
Mean = 38.6 ± 9.51				

The highest proportion (31.2%) had been serving as a teacher for less than a year to 5 years, this was followed by 23% who had been working as a teacher from 5 to 9 years.

A majority (66.7%) of the respondents professed to be Catholics while the rest are non-Catholics. Two-thirds (65.6%) had a college degree while the rest had completed either a master’s or a doctorate degree. More than a fifth of the respondents (22.3% to 25.6%) taught Grades 7 to 9 pupils. Around 14% to 15% taught Math, Science and MAPEH (Music, Arts, Physical Education and Health). Much smaller proportions taught other subjects like TLE (Technology and Livelihood Education), English, Social Studies, and Values Education.

A little more than 60% of the respondents said they have at least a woman of reproductive age group living in their households, but only 5% had a pregnant woman in the same household at the time of interview. A little over a third (34%) had a monthly family income that ranged from PhP 20, 000 to 29, 000 (400 to 580 USD).

### Knowledge on the sources of information

Almost everybody claimed to have heard of Zika Virus Disease and a quarter of the respondents said that they heard about Zika as early as 2014.

Table 3 shows that tri-media/triple media which includes TV, radio, and print (87.3%), was the most frequent source of information about Zika Virus. This was followed by social media (28.1%) and to a lesser extent by interpersonal communication channels like family, friends, and health workers.

**Table 3:** Distribution of respondents according to age group and sex (n = 609).

Source of Information about Zika Virus*	Frequency	Percentage
Tri-media/ public Announcement	532	87.3
Social Media	171	28.1
Family/ Friends/ Neighbors	60	9.9
Health workers/ Door-to-door Campaign/ Pharmacy	59	9.7
International and Local Organization	27	4.4
Local Healer/ Religious Leaders	3	0.5
No Response	14	2.3

*Multiple response

### Knowledge on the modes of transmission of ZIKV Disease

A very high proportion (80.3%) mentioned that Zika Virus Disease is transmitted through a mosquito bite. This was followed by, to a lesser extent, mother to child transmission, through blood transfusion, and from a dirty environment. It is noteworthy that 12.3% did not know how the disease is transmitted and 8% who mentioned coughing and sneezing as a mode of transmission (Table 4).

**Table 4:** Frequency and percentage distribution of respondents according to their knowledge on how a person gets Zika (n = 609).

How does a person get Zika?*	Frequency	%
Mosquito Bite**	489	80.3
From Mother to Child Transmission**	139	22.8
From a Blood Transfusion**	104	17.1
From a dirty environment	88	14.4
Through coughing and sneezing	48	7.9
Sexual Intercourse**	48	7.9
From Breast Milk	29	4.8
Drinking of dirty water	27	4.4
Bathing in polluted water	14	2.3
I don’t know/ Other answers/ No response	75	12.3

*Multiple response

**Correct answer

### Knowledge on signs and symptoms of ZIKV Disease

Table 5 shows that the respondents were able to identify fever (64.7%) and headache (38.6%) as a sign and a symptom of Zika Virus Disease, respectively. A much fewer respondents identified other signs and symptoms of the said disease. It is noteworthy that 28% admitted not knowing the signs and symptoms of Zika Virus Disease.

**Table 5:** Frequency and percentage distribution of respondents according to their knowledge on the signs and symptoms of Zika Virus Disease (n = 609).

Signs and Symptoms	Frequency	%
Signs*		
Fever	394	64.7
Hemorrhage/ Bleeding	96	15.8
Rashes	88	14.4
Conjunctivitis (Red Eyes)	50	8.2
Symptoms*		
Headache	235	38.6
Joint Pain	176	28.9
Sickness	152	25.0
Diarrhea	35	5.7
I don’t know/ No Response/ Other answers	171	28.0

*Multiple response

### Knowledge on preventive measures against ZIKV Disease

The respondents were asked about their knowledge on the preventive measures against Zika Virus Disease. The responses were logically grouped into three generic categories: 1) environmental management and/or vector control measures; 2) use of self-protection; and 3) other beliefs or misconceptions. Table 6 reveals that 55.7% and 54.5% of the respondents identified cleaning/scrubbing of storage containers and house environment as preventive measures, respectively. Also, 50.4% identified use of mosquito repellants or sprays as another preventive measure. Self- protection measures were also mentioned to a much lesser extent. It is worth to note that there were some who had misconceptions about Zika Virus Disease preventive measures.

**Table 6:** Frequency and percentage distribution of respondents according to their knowledge of preventive measures against Zika Virus Disease (n = 609).

Preventive Measures*	Frequency	%
***Through environmental management/vector control***		
Clean/ scrub water storage containers	339	55.7
Clean household environment	332	54.5
Remove standing/ stagnant water	253	41.5
Put screens on windows or doors	196	32.2
Use of larvicides	179	29.4
Spray or fumigate my home	128	21.0
***Use of self-protection***		
Use mosquito repellant or spray on your body	307	50.4
Use mosquito net at night	265	43.5
Wear covering clothes like long pants and shirts with long sleeves	162	26.6
Use mosquito coil/ light fires to keep mosquito away	113	18.6
Use mosquito net during the day	85	14.0
Use a condom/ have my partner use a condom in all sexual relations	37	6.1
Abstain from sexual intercourse	26	4.3
***Other beliefs/Misconceptions***		
Pray to God	77	12.6
Drink clean water	72	11.8
Wash in clean water	64	10.5
I don’t know/ Other answers/ No response	103	16.9

*Multiple response

In terms of their knowledge on the possible complications of ZVD, the respondents were asked about their understanding of microcephaly (Table 7). Thirty percent (30%) said that it is a condition where the brain becomes smaller in size. A majority of the respondents (62%) admitted that they did not know what microcephaly was.

**Table 7:** Frequency and percentage distribution of respondents according to their knowledge on what is microcephaly (n = 609).

What is microcephaly?	Frequency	%
Brain/head becomes smaller	182	29.9
It is a neurologic abnormality	11	1.8
It is caused by a virus and/or bacteria	10	1.6
Others: (disease, enlarged brain, hot headed)	31	5.1
I don’t know/ Other answers/ No response	375	61.6
Total	609	100.00

### Respondents’ perceptions of their susceptibility and severity of Zika Virus Disease

The respondents were likewise asked about their perceptions related to their susceptibility to and the severity of Zika Virus Disease in their community. Table 8 shows that 61% said that it is possible for them to acquire the disease, and that the virus is deadly or can cause death (55%). Nearly half (43.3%) of the respondents claimed that their risks of getting Zika Virus Disease in the next six months ranges from moderate to high. Only 49.8% mentioned that the virus can cause disability to newborns.

**Table 8:** Frequency and percentage distribution of respondents according to perception of their susceptibility to and severity of Zika Virus Disease (n = 609).

Perception of Susceptibility and Severity of Zika Virus Disease*	Frequency	%
They can acquire the Zika Virus Disease	369	60.6
Zika Virus is deadly or can cause death	335	55.0
Zika Virus can cause disability to newborns	303	49.8
Possibility to acquire Zika Virus in their Community	283	46.5
With medium to high perception of risk that they might get Zika in the next 6 months	264	43.3
Zika Virus disease can cause disability to the elderly	135	22.2
Had known someone who had Zika Virus Disease in their community	3	0.5

*Multiple response

### Respondents’ attitudes towards ZIKV Disease

The respondents’ attitudes towards Zika Virus Disease were also assessed using a four-point Likert Scale. Table 9 reveals that more than 88% agreed that a private and a public doctor can effectively treat a person infected with Zika virus. Also, more than two-thirds disagreed with statements that a pharmacy or local drug vendor, and local healers can effectively treat Zika. A high 80.1% agreed with the belief that all pregnant women should be tested for Zika. Even a higher proportion (89.6%) agreed that they would consider vaccination against Zika, if it is available and 89.5% believe that a vaccine can prevent the spread of Zika. However, there was an ambivalence among the respondents about their attitudes towards the use of artificial contraceptive methods to prevent pregnancy and Zika Virus Disease. Majority (72%) of the respondents disagreed with the use of abstinence and non-use of modern family planning methods to prevent pregnancy and Zika. Only half agreed with the statement that women should use condom or have her partner use a condom in all sexual encounters to avoid Zika. The respondents were also divided as far as statements related to stigma and discrimination related to Zika were concerned.

**Table 9:** Percentage distribution of respondents’ attitudes towards Zika Virus Disease (n = 609).

Attitudes Towards Zika Virus Disease*	Agree (%)	Disagree (%)
Zika is an important issue/ problem in my community	91.9	6.4
***Attitudes Towards Treatment***		
A private doctor can effectively treat a person with Zika	89.8	8.0
A public hospital can effectively treat a person with Zika	88.0	9.7
A pharmacy or local drug vendor can effectively treat a person with Zika	29.1	68.8
A local healer *(Hilot)* can effectively treat a person with Zika	20.0	77.8
***Attitudes Towards Testing for Zika***		
I believe that all pregnant women should be tested for Zika	80.1	17.6
If I have fever now, I would consider being tested for Zika	66.7	30.5
***Attitudes Towards Prevention***		
I would consider having vaccination against Zika if it is available	89.6	7.6
I believe that a vaccine can prevent the spread of Zika	89.5	7.7
Women should use condom or have her partner use a condom in in all sexual encounter to avoid Zika	49.0	47.4
Women should use contraceptive pills to avoid getting Zika	48.2	48.7
During this time, women should avoid getting pregnant because of Zika	44.3	52.9
Women should not use injectable contraceptives to avoid getting Zika	37.8	58.0
Women should use long acting reversible contraception (IUD, implants, etc.)	37.1	59.8
Women should only resort to abstinence from sexual intercourse in order to avoid Zika	24.0	72.0
Women should not use other modern family planning methods	22.3	74.9
***Attitudes Towards Stigma***		
If a person gets Zika, he/she is discriminated or stigmatized because of it	48.4	48.4
If a person gets Zika, his/ her family is discriminated or stigmatized because of it	47.2	50.0
If somebody in my family were to get Zika, I would want it to remain private or a secret	32.3	64.6

*does not add up to 100% due to missing data

### Respondents’ practices related to cleaning/scrubbing water sources/ containers

The respondents were asked as to when was the last time they cleaned or scrubbed their water source which could serve as a breeding for mosquito vectors (Table 10). Half of them (54%) said within the last week, followed by those who cleaned their water source more than a week ago (19%).

**Table 10:** Frequency and percentage distribution of respondents as to the last time they cleaned/ scrubbed their water source/storage unit/water containers (n = 609).

	Frequency	%
Within the last week	328	53.9
Over a week ago	113	18.6
Last month	20	3.3
Two to six months ago	22	3.6
Seven to twelve months ago	5	0.8
I don’t know/ Other answers/ No Response	96	15.8

Table 11 shows the proportion of correct knowledge on transmission of Zika virus by sociodemographic characteristics. It appears that respondents who are 39 years old and older, males, with bachelor’s degree only, with religion other than Catholic, with above P 30,000 monthly family income, residing in urban areas, living in close proximity to a health center, and teaching health-related subjects have a higher correct knowledge on the transmission of Zika virus by mosquito and sex. However, statistical association using Pearson’s Chi-square test did not reach statistical significance.

**Table 11:** Association of knowledge on Zika virus transmission and sociodemographic characteristics.

	Knowledgeable^a^ No (%)	p value^b^
Age Group		
Less than 39 years old	25 (7.2)	1.000
39 and older	17 (7.6)	
Sex		
Female	32 (7.2)	0.667
Male	11 (8.3)	
Highest Educational Attainment		
Bachelor’s degree	33 (8.3)	0.229
With at least post-graduate units	8 (5.3)	
Religion		
Catholic	30 (7.4)	0.944
Non-Catholic	13 (7.6)	
Monthly Family Income		
≤ P 30,000 (less than US$ 572)	30 (7.4)	0.574
Above P 30,000	13 (7.6)	
Location of Residence		
Urban	40 (8.2)	0.183^c^
Rural	2 (3.3)	
Travel time from house to nearest health facility		
Within 5 minutes by walking	21 (9.4)	0.255
Within reach by at least 10 minutes by vehicle	22 (6.7)	
Subject taught		
Health-related subjects	15 (8.6)	0.415
Non health-related subjects	29 (6.7)	

^a^ Knowledgeable–answered 2 knowledge items on Zika transmission correctly

^b^ P–value of a Pearson’s Chi-squared test unless otherwise specified.

^c^ Fisher’s exact test; for analysis of small numbers (at least one cell containing < 5 counts)

Table 12 shows that females, those with bachelor’s degree only, non-Catholics, with above P 30,000 monthly family income, residing in urban areas, living in close proximity to a health center, and teaching health-related subjects appear to have a more positive attitude toward Zika. However, only income and location of residence were found to be significantly associated with a positive attitude toward Zika as indicated with p–value which is equal to 0.05 or less.

**Table 12:** Association of attitudes toward Zika virus and sociodemographic characteristics.

	Positive Attitude^a^ No. (%)	P–value^b^
Age Group		
Less than 39 years old	167 (63.7)	0.223
39 and older	95 (36.3)	
Sex		
Female	202 (72.1)	0.646
Male	64 (22.9)	
Highest Educational Attainment		
Bachelor’s degree	187 (72.8)	0.923
With at least post-graduate units	70 (27.2)	
Religion		
Catholic	184 (68.7)	0.581
Non-Catholic	84 (31.3)	
Monthly Family Income		
≤ P 30,000 (less than US$ 572)	204 (77.9)	0.001*
Above P 30,000	183 (22.1)	
Location of Residence		
Urban	218 (86.2)	0.05*
Rural	35 (13.8)	
Travel time from house to nearest health facility		
Within 5 minutes by walking	95 (37.5)	0.218
Within reach by at least 10 minutes by vehicle	158 (62.5)	
Subject taught		
Health-related subjects	77 (27.5)	0.321
Non health-related subjects	203(72.5)	

^a^ Positive attitude–disagreed with 2 negatively-phrased attitude statement toward Zika

^b^ Fisher’s exact test; for analysis of small numbers (at least one cell containing < 5 counts)

*statistically significant

## Discussion

Since 2012, the Philippines has reported only a few cases of Zika Virus Disease but this situation should not lead to complacency among the local health authorities. With the increase in tourist arrivals and returning Overseas Filipino Workers (OFWs) who may come from endemic areas, and the changing ecology that favors mosquito breeding, the probability of ZIKV outbreaks is always present. Considering the unavailability of vaccine against ZIKV disease, health promotion remains to be the most cost-effective strategy in reducing mosquito vector population density and human-vector contact, thereby reducing the risk of ZIKV transmission. This study was the first in the Philippines that aimed at determining the knowledge, attitudes, and practices of public secondary school teachers related to Zika Virus Disease. The findings can serve as a basis for the development and evaluation of health education interventions in the future. Through this, the role of teachers as the primary and credible source of information of students on health topics can be optimized.

A high level of awareness on ZIKV was found among secondary school teachers as indicated by their self-report that almost everyone has heard about the disease. This can be attributed to the attention given to Zika by local news networks following the reports of outbreaks in South America. The finding is consistent with other studies which found a similarly high level of awareness (77% to 91%) on Zika among different population groups [13, 14, 18, 19]. The reported high level of awareness is corroborated by the findings that tri-media—TV, radio, and print, (87%) was the most frequent source of information about Zika [13, 18, 19]. This level of awareness was more or less consistent across various population groups (medical residents, women, pregnant women and immigrants) who participated in previous studies. Despite the high level of awareness, several participants in this study still had some misconceptions about ZIKV disease such as transmission through coughing and sneezing, drinking of dirty water, and bathing in polluted waters. Despite its widespread popularity and use vis-à-vis the relatively young mean age of the study respondents, only 28% of the participants identified social media as their source of information on ZIKV. This is lower compared to the 43.7% among the study respondents in Qatar [18] but higher among pregnant women respondents in Brunei Darussalam [26]. This may imply that information related to ZIKV was either not adequately circulated in the social media or the respondents was not able to access this information. A systematic review in 2013 showed that social media increases accessibility and widens access to health information. Social media can be an important platform for disseminating health information if properly used and monitored [27].

A high proportion (80%) of respondents knew that Zika can be transmitted through mosquito bites, which may a indicate good knowledge on this particular mode of transmission. This can be attributed to high level of awareness of the respondents on Zika Virus Disease mode of transmission as influenced by the tri-media (TV, radio and print). Other studies corroborated such finding [15, 19, 28]. One study showed that only around 58% of their participants mentioned that Zika was transmitted by any mosquito bite, however, the same study also mentioned that nearly 20% said that the virus is transmitted through *Aedes aegypti* bite [28]. This study was not able to combine all the responses related to transmission by mosquitoes which, when combined, could have reached 78%. A related study in China used two methods of data collection, one was through internet survey which had a very high proportion of respondents (95.2%) who knew about ZIKV transmission via mosquitoes while those recruited through paper-based interview only had 67.3% [29]. Those who responded through the online survey had a much higher level of health literacy because they were much younger and had a higher level of education. It is also possible that respondents could have checked on-line references during the survey.

Only 8% of the respondents identified sexual intercourse as a mode of transmission of ZIKV compared to 34.6% to 59% of respondents in other studies [15, 16, 19, 30] but consistent with a study finding among Ecuadorian adults [31]. This is supported by the findings where only 10% of the respondents said that condom use and abstinence were measures which could prevent ZIKV transmission. Moreover, less than half of the respondents agreed with attitude statements such us women should use condoms or have her partner use a condom in all sexual encounters to avoid Zika (44.3%) and that, in times where there is active viral transmission, women should avoid getting pregnant because of Zika (49%) [32]. These findings are worrisome because this poor knowledge reinforced by a negative attitude could lead to an undesirable practice which might put women and their future children at risk for ZIKV disease and its complications. These findings may be explained by the fact that ZIKV shares the same mosquito vector as that of dengue. Since the media reports almost always reported the two vector-borne diseases together, there is the possibility that the teachers recalled the transmission through mosquito bite more often than through sexual intercourse. This poor knowledge which may increase the respondents’ susceptibility to ZIKV can be compounded by the number of returning Filipino overseas workers, e.g. seafarers, who might come from areas where there is local transmission of the virus [7].

While dengue has already been integrated in the curriculum, topics on ZIKV is not yet part of secondary school subjects due to lack of teachers’ training, low confidence in teaching the topic, and the unavailability of teaching-learning resources which they can use in the classrooms.

Only 23% of the respondents knew that the Zika virus is transmitted from mother to child. Such finding is very low considering the respondents’ high level of awareness and knowledge that Zika Virus is transmissible through mosquito bite. The possible explanation is that Zika became more commonly known as a vector-borne disease rather a disease transmitted through the perinatal route. It was only recently that people learned about the possibility of mother to child transmission. In other studies, only 35.0% to 56.8% of the respondents knew that Zika Virus can be transmitted from mother to child [13, 15, 18]. Nevertheless, the low proportion of response in the present study is still higher compared with the 14% found by Aidoo-Frimpong, et. al., among the general Ecuadorian population [31]. This may be attributed to the higher educational attainment of teachers, i.e. at least college graduate, who participated in this study.

There were studies which found a much higher proportion of respondents who knew the possibility of a mother to child transmission. In the study of Borges, et. al., they found out that almost every respondent who had ever heard about Zika was aware of the relationship of the virus with congenital syndrome. This study was related to Zika outbreaks in Brazil which had infected more than 200,000 people and generated 2,753 cases of confirmed congenital syndrome. The magnitude of the problem in Brazil could have contributed to this high level of knowledge on mother to child transmission. Moreover, Borges’ respondents were recruited from pregnant and non- pregnant women who had scheduled medical and nursing consultations at the health facilities and would have probably received more information about Zika compared to other population groups [12]. Arief et. al observed that more than 80% of their survey respondents said that pregnant women are at high risk for Zika Virus attesting to their high level of knowledge of the relationship between Zika and pregnancy [19]. Mouchtouri et. al found that only 38% and 47% of their respondents said that women who are pregnant are at risk of miscarriage and having a child with microcephaly should they be infected by Zika Virus, respectively [13].

Knowledge on signs and symptoms is important in the early recognition and prompt referral of cases of Zika to the health facilities. A majority of the participants had mentioned fever (65%), followed by headache (38.6%), joint pain (29%), rashes (14.4%) and conjunctivitis (8.2%). A quarter (28%) of the respondents did not know about the signs and symptoms Zika, which is alarming because this could delay diagnosis and prompt treatment, and could contribute to the spread of the virus. Poor knowledge on the recognition of signs and symptoms maybe be due to the content of some health communication materials that focuses on the modes of transmission, prevention, and control but less on clinical signs and symptoms of ZIKV Disease. These findings did not vary much with those found by Samuel in their study among immigrants in New York City [30]. It was difficult to compare the signs and symptoms reported in other studies because of varying ways of measuring and reporting knowledge of the signs and symptoms of ZVD. Arief et. al reported that 73.8% of their respondents knew that joint pain, fever, conjunctivitis, and skin rash as signs and symptoms of ZIKV [19]. Plaster et. al reported 71% and 46% of their respondents mentioned fever and skin rash as signs of ZIKV disease respectively [15].

A very high proportion of respondents knew that the virus is transmitted through mosquito bites. However, only half of the participants identified vector control as a preventive measure. This is an area that needs to be strengthened to prevent ZIKV disease outbreaks. With the absence of an effective cure and vaccine against ZIKV, health education, as well as environmental control measures, remains to be the main strategy as far as prevention and control program is concerned. This result was better compared to the findings of a study done among Colombian respondents, in which only 30% identified elimination of breeding sites and the use of mosquito repellants as important preventive measures [28].

Only 30% of the respondents in this study were able to describe microcephaly. A high proportion (62%) did not know what microcephaly is. The same was noted in another study, in which only half of their respondents said that ZIKV can cause disability to newborns, which could be referring to microcephaly. The high proportion of respondents who admitted not knowing what microcephaly is or that Zika Virus is not associated with microcephaly can lead people to practice risky behaviors because they did not know that having a small brain is a complication of ZIKV infection. This finding is similar with what Plaster et. al found out in their study among university students in the USA [15]. On the other hand, another study found out a very high proportion (95%) of community members in the USA knew the clear link between Zika Virus and microcephaly [30].

Among the respondents, 60% and 55% agreed with the statements such as ‘they can acquire the Zika Virus Disease’ and ‘ZIKV is deadly and can cause death, respectively. Half (49.8%) of the respondents felt that there is a possibility to acquire the ZIKV in their community. This finding is an indication of a moderate level of perceived susceptibility and severity of ZIKV among the teachers probably because: 1) they have not encountered a case of ZIKV infection in person, and 2) they know that the number of ZIKV cases in the Philippines is still low. Hence, their perceived level of susceptibility to ZIKV disease is at a moderate level. This is supported by a study done among residents of Selangor, Malaysia where only half felt that their country is at high risk for spread of ZIKV and 33% who had ever thought they could get the virus [19]. Nearly half (47.0%) of respondents in a study in China and 47.5% in Ecuador were worried about contracting Zika and half of the adult respondents in an Ecuadorian study felt at risk to contracting ZIKV [29, 31]. This level of perceived susceptibility is much higher compared to those found by other studies in the US [15], Ecuador [17], and Malaysia [19].

A very high proportion of participants (92%) believed that Zika is an important issue in their community and 90% perceived that a private doctor and a public hospital can effectively treat a person with Zika. This finding reveals that the participants recognize the importance and threat caused by ZIKV and positive health beliefs related to Zika treatment which can influence their positive health seeking behaviors.

The respondents’ low level of perceived susceptibility to ZIKV aligns well with their poor preventive behaviors. Only half admitted to have cleaned their water containers or water sources within the last week and that 16% of the respondents did not know or provided no response to this important practice of removing potential mosquito breeding sites [28]. In the absence of effective drugs and vaccine against Zika, regular cleaning of water containers to prevent breeding of mosquito vector is critical in preventing disease outbreaks. This practice of cleaning water containers is much lower compared to a community in Washington, USA whose residents are more aware and have a better Zika-preventive practice [16]. This finding is worrisome since open water containers and stagnant water sources are potential mosquito breeding sites which can give rise to vector-borne epidemics such as ZVD.

This study found that respondents who are 39 years old and older, males, with bachelor’s degree only, non-Catholics, with above P 30,000 monthly family income, residing in urban areas, living in close proximity to a health center, teaching health-related subjects seem to be a little more knowledgeable about transmission of Zika virus through mosquito and sex. However, statistical association using Pearson’s Chi-square test did not reach statistical significance. Other studies found that knowledge on Zika transmission was not associated with social status/income [12, 19] but was associated with residence (urban/rural) [19]. Others have reported statistically significant association of general knowledge on Zika with older respondents [18, 26]. The present study shows that respondents who had attained a bachelor’s degree were found to have a better knowledge on Zika compared to those who had at least postgraduate units [26]. While this appears to be counter-intuitive, however, it could be due to the fact that those with bachelor’s degree belong to a younger subset of population whose access to social media with health-related information is greater [27, 29]. Other studies confirmed the significant association of education and knowledge [19, 26, 27, 33, 34]. Mixed results have been found between knowledge on Zika and sex [18, 27]. Residing in urban areas and proximity to health centers could be associated with knowledge about Zika as these imply better access to health-related information. However, due to the low number of participants with sufficient knowledge on Zika transmission, the association did not reach statistical significance.

This study also found that family income and location of residence were statistically associated with having a positive attitude towards the disease. Association of monthly family income with attitude towards ZVD mean that people with higher socioeconomic status have better access to health services and information sources about Zika, and therefore have a higher health literacy [35, 36]. The association between urban residence and having a positive attitude toward Zika could be still be related to a greater exposure to health messages and access to health services in urban areas. This finding is consistent with a study in Selangor, Malaysia where residents living in rural areas had a favorable attitude towards Zika [19]. Some studies did not include location of residence as part of the sociodemographic profile [14, 15, 26, 28] while others use a different measure for attitude [11, 30].

The lack of statistically significant association between the socio-demographic characteristics and knowledge on Zika transmission as well as attitude towards Zika (except for income and type of residence) should be viewed with reference the low number of participants with sufficient knowledge and attitude on Zika transmission that reduced the power of the test to detect statistically significant associations.

## Conclusion

Teachers who participated in this study had a good level of knowledge on the transmission of ZIKV through mosquito bite but poor knowledge about mother to child transmission, transmissions through sexual intercourse and breastfeeding, complications of the infection, and preventive practices. Their perception towards susceptibility to and severity of ZIKV is low which could probably be due to the absence of an actual encounter with a Zika patient and the low number of reported cases in the Philippines. Attitudes towards the effectiveness of doctors in treating Zika is high but some still believe that local healers can effectively treat Zika. Respondents’ knowledge and attitudes toward prevention of sexual transmission is low. There was ambivalence in terms of attitude towards social stigma related to ZIKV. Given that the ingredients of a possible Zika epidemic is present in the Philippines, the education materials that can be developed for teachers should include topics that will improve their perceptions towards their susceptibility and severity of ZIKV, their knowledge on sexual mode of transmission, how to prevent the virus from being transmitted through sexual intercourse or mother to child, and the practice of regularly cleaning open water containers which can serve as potential mosquito breeding sites. Correcting misconceptions such as transmission through coughing and sneezing, drinking of dirty water and bathing in polluted water should also be included in the educational materials to prevent spreading these misconceptions to learners. Learners look at their teachers as their role models and a reliable source of knowledge, hence, the latter’s competency on Zika should be no less than satisfactory. Social media can also be used as a supplementary channel for in-service training for teachers since the results imply that those who only had bachelor’s degree appear to be younger respondents access this type of media for health-related information. This study provided baseline data from which the effects of future curricular interventions on teachers’ knowledge, attitudes and practices related to Zika virus can be compared against.

Income and location of residence were found to be significantly associated with attitude towards Zika virus and ZVD. These highlight the role of a social determinant, i.e. income, in influencing health-related attitudes and overall health.

The above results and conclusion should be taken in the light of the study’s limitations. Despite the explanation of the potential contribution of the study to teachers, the teachers’ response rate was reported at 53%. Self-selection bias might have affected the results of the study since it was possible that study participants might have a different set of characteristics than non-study participants. The use of self-administered questionnaire might also have introduced some information bias but this was minimized by anonymizing the tool and adding response options.

## Supporting information

S1 Dataset (XLSX)
